# KIF2C is essential for meiosis and manchette dynamics in male mice

**DOI:** 10.3389/fcell.2025.1523593

**Published:** 2025-03-27

**Authors:** Ryua Harima, Mayu Kishinami, Kenshiro Hara, Kentaro Tanemura

**Affiliations:** ^1^ Laboratory of Animal Reproduction and Development, Graduate School of Agricultural Science, Tohoku University, Sendai, Miyagi, Japan; ^2^ Laboratory of Reproductive Technology (Repro-SOLEIL), Graduate School of Agricultural Science, Tohoku University, Sendai, Miyagi, Japan

**Keywords:** KIF2C (MCAK), microtubule dynamics, meiosis, chromosome alignment, manchette, male infertility

## Abstract

In gametogenesis, microtubules undergo dramatic changes known as microtubule dynamics, and which is important for fertility both male and female. In spermatogenesis, spindle microtubule dynamics occur during meiosis and manchette microtubule dynamics occur in elongated spermatids. In oogenesis, spindle microtubule dynamics occur during meiosis. The microtubule depolymerization protein kinesin-13 family (KIF2A, KIF2B, and KIF2C) plays an important role in microtubule dynamics, and KIF2C is a well-known microtubule depolymerization factor in mitosis. Although the function of KIF2C in mitosis has been extensively studied, its role in meiosis remains unclear. Additionally, the role of microtubule dynamics in manchette formation remains unclear. We generated germ cell-specific *Kif2c* conditional knockout (*Kif2c* cKO) mice to elucidate KIF2C function in germ cells. *Kif2c* cKO male mice showed chromosomal misalignment at meiotic metaphase, abnormal manchette morphology and delayed manchette disassembly, which led to a significant increase in apoptosis. Furthermore, *Kif2c* cKO male mice were completely infertile. Therefore, KIF2C plays an important role in chromosomal alignment in male meiosis and in manchette dynamics in elongated spermatids. In contrast, *Kif2c* cKO female mice were sufficiently fertile, and only minor defects were observed in chromosome alignment in meiosis. This study demonstrates, for the first time, that KIF2C is important for microtubule dynamics of spermatogenesis to achieve male fertility, but not for female fertility.

## 1 Introduction

In gametogenesis, microtubules dramatically change their shape in many processes, collectively known as microtubule dynamics, in which microtubules undergo repeated polymerization and depolymerization (Mitchison and Kirschner, 1984). There are three main processes involved in spermatogenesis: microtubule dynamics in spindle formation and chromosome congression and segregation in meiosis, microtubule dynamics in manchette formation during spermiogenesis, and microtubule dynamics in flagellum formation (O'Donnell and O'Bryan, 2014). In oogenesis, microtubule dynamics occur during meiosis (Coticchio et al., 2015).

In meiosis, the spindle is formed at prometaphase (Helmke et al., 2013). The spindles attach to the kinetochores at prometaphase (spindles that attach to the kinetochores are called K-fibers) (Valdez et al., 2023), and chromosomes align at the equator by the tension generated in the K-fibers in metaphase (Cross and McAinsh, 2014). When the spindle microtubules have correctly attached to kinetochores and aligned to the equator, they proceed to anaphase (Musacchio and Salmon, 2007). The manchette is the skirt-like structure of microtubules that appears transiently surrounding the head of elongated spermatids (Kierszenbaum and Tres, 2004; O'Donnell and O'Bryan, 2014; Russell et al., 1991). In mice, the manchette is important for the head shaping and flagellum formation in elongated spermatids (Kierszenbaum et al., 2011; Russell et al., 1991) and the proteins necessary for these processes were transported along the manchette (Kierszenbaum, 2002; Kierszenbaum and Tres, 2004; Lehti and Sironen, 2016). Intensive microtubule dynamics occur in the manchette, whereas the regulatory mechanisms of microtubule dynamics are unclear. Flagella are microtubule structures unique to mammalian sperm, and the sperm is motile using flagellar propulsion (Gibbons, 1981; Lindemann and Lesich, 2016). The sperm flagella have long microtubule structure called the 9 + 2 axoneme consisting of two central microtubules surrounded by nine peripheral microtubules (Luck, 1984; Porter and Sale, 2000).

Proper microtubule depolymerization is important for microtubule dynamics. The kinesin 13 family is responsible for microtubules depolymerization (Desai et al., 1999; Signor et al., 1999). There are three members of the kinesin-13 family: KIF2A, KIF2B, and KIF2C (also known as MCAK) (Cross and McAinsh, 2014). KIF2C is the best-characterized member of the family responsible for microtubule depolymerization in mitosis. KIF2C localizes to centromeres and spindle pole in mitosis, and modulates the tension generated by spindle microtubules via Aurora kinase B regulation (Ems-McClung et al., 2007; Ganem et al., 2005; Waters et al., 1996; Zhang et al., 2007). In previous studies, it was found that KIF2C depletion or inhibition causes chromosome misalignment and segregation errors in mitosis (Bakhoum et al., 2009; Ganem et al., 2005; Kline-Smith et al., 2004; Maney et al., 1998; Wordeman et al., 2007). In female meiosis, KIF2C disruption causes chromosome misalignment and segregation errors, leading to meiotic arrest (Eichenlaub-Ritter et al., 2010; Illingworth et al., 2010; Vogt et al., 2010). In contrast, another study showed that KIF2C is involved in chromosome alignment and segregation but is not necessary for meiosis completion in females (Vogt et al., 2010), and the detailed function of KIF2C in female meiosis is unclear. In addition, the effect of KIF2C on fertility *in vivo* using individuals has not been assessed. In male meiosis, very few studies have focused on KIF2C, and the localization analysis using immunostaining have only been conducted (Parra et al., 2009; Parra et al., 2006). In previous study, KIF2C was found at centromeres in metaphase Ⅰ and Ⅱ in spermatocytes, and was regulated its loading at centromeres by Shugoshin 2 (Parra et al., 2009; Parra et al., 2006). In addition, KIF2C forms ring structures with SCP3 or Aurora kinase B surrounding and beneath kinetochores at metaphase Ⅰ and Ⅱ, which is a novel centromeres domain in male meiosis (Parra et al., 2006). However, there are no studies investigating the function of KIF2C in male meiosis, thus its function is unclear. As noted above, KIF2C shows a specific expression pattern in meiosis, so it is possible that KIF2C has a unique function in meiosis, not found in mitosis. Therefore, the analysis of KIF2C focusing on meiosis is required. Furthermore, the involvement of microtubule dynamics in the manchette and flagella, other than meiosis, remains unknown. Interestingly, *Kif2c* mRNA is highly expressed in the testis (https://www.ncbi.nlm.nih.gov/gene/73804). We hypothesized that KIF2C plays an important and distinctive role in the microtubule dynamics during many processes of spermatogenesis. To explore the function of *Kif2c* in germ cells, we attempted to generate *Kif2c* knockout mice. Since *Kif2c* global knockout mice are preweaning lethality (International Mouse Phenotyping Consortium; https://www.mousephenotype.org/data/genes/MGI:1921054), we generated germ cell-specific *Kif2c* conditional knockout mice and aimed to elucidate the function of KIF2C in microtubule dynamics in gametogenesis.

## 2 Materials and methods

### 2.1 Animals

*Kif2c* ^flox/flox^ mice of C57BL/6N background and Ddx4-Cre (Vasa-Cre) transgenic mice (#:006954, The Jackson Laboratory) of FVB background were used (Gallardo et al., 2007). C57BL/6N mice were purchased from SLC and all mice were housed in a 12-h light/dark cycle at 24 ± 1°C, and 60 ± 10% humidity. The mice had free access to food (MF; Oriental Yeast Co., Ltd.) and water. All methods were performed in accordance with relevant guidelines and regulation. Animal handling and experiments were conducted according to protocols approved by the Tohoku University Institutional Animal Care and Use Committee (2019noudou-003-02, 2019noukumikae-030-06).

### 2.2 Generation of germ cell-specific *Kif2c* conditional knockout mice

Germ cell-specific *Kif2c* conditional knockout mice (*Kif2c* cKO) of the C57BL/6N strain were generated using *i*-GONAD, as previously reported (Gurumurthy et al., 2019). To generate *Kif2c* ^flox/flox^ mice, firstly, one loxP site was inserted in an intron between exons 1 and 2 (That mice call loxP site 5′- knock-in mice) using the *i*-GONAD method. Next, loxP site 5′- knock-in mice were mated with each other, and the other loxP site was inserted in the intron between exon 10 and exon 11 into its zygotes at day 0.7. These mice harbored a loxP site flanking exons 2 and 10 and were thus called *Kif2c* ^flox/flox^ mice. The crRNA (Integrated DNA Technologies) and ssODN (Integrated DNA Technologies) were designed with the following sequences to insert the loxP site between exon 1 and exon 2:5’- AAATTTACATTTACCTCGGA -3’ and 5’- CCAGGGCTACACAGAGAAACCCTGTCTCGAAAAAACAAACAAACAAACAAACAAAAAAAAACAAAAACAAAACAAAACAAATTTCCCTTCCATAACTTCGTATAGCATACATTATACGAAGTTATGAGGTAAATGTAAATTTGAAGTCTTTAGGATCCTAA -3’. The crRNA and ssODN were designed with the following sequences to insert the loxP site between exons 10 and 11:5’- CATACGACATATTTTCGGCC -3’ and 5’- AGGCCAGCCTAGGCTATAGTGTGAGATCCTAATTATGACAGACATTCTATGTAGTTCACATACGACATATTTTCGATAACTTCGTATAGCATACATTATACGAAGTTATGCCTGGTTTTCTTTTGCCCTTTGTAATACATCCTGAGCAGTGGGGTCAGAGGAGGGGCTGTTTGGCTTAAAGTCT -3’. For the preparation of gRNA, crRNA and tracrRNA (Integrated DNA Technologies) were annealed at 94°C for 2 min, mixed with ssODNs and Cas9 nuclease, and injected into the oviduct on day-0.7 pregnant mice. The primer sequences used for genotyping were as follows: to detect the loxP site between exon 1 and exon 2, Fw1:5’-CCACTGCCTGGCTGTTTGTA-3’ and Rv1:5’-CCACTGCCTGGCCAAACTTA-3’. To detect the loxP site between exons 10 and 11, Fw2:5’-GAGGTTTGGAGGTGCAGTCT-3,’ Rv2:5’-ACCCCACTGCTCAGGATGTA-3’ were used.

The PCR product from the mutant DNA was sequenced using Sanger sequencing to confirm successful insertion. *Kif2c* ^flox/flox^ mice were crossed with Ddx4-Cre (Vasa-Cre) transgenic mice (#006954, Jackson Laboratory) to generate *Kif2c* ^flox/+^; Vasa-cre mice. Furthermore, *Kif2c* ^flox/flox^ mice were crossed with *Kif2c* ^flox/+^; Vasa-cre mice to generate *Kif2c* ^flox/-^; Vasa-cre mice. *Kif2c* ^flox/-^; Vasa-cre mice were called germ cell-specific *Kif2c* conditional knockout (*Kif2c* cKO) mice in this study. The primer sequences used for genotyping to detect Cre were as follows: Fw: 5’- CACGTGCAGCCGTTTAAGCCGCGT-3’, Rv: 5’- TTCCCA-TTCTAAACAACACCCTGAA-3’. *Kif2c* ^flox/+^; Vasa-cre mice, *Kif2c* ^flox/-^; Vasa-cre mice and *Kif2c* ^+/−^; Vasa-cre mice were crossed with C57BL/6N mice at least three times in this study.

### 2.3 Mouse tissue collection

Mice were euthanized by cervical dislocation under anesthesia and the tissues were collected. We used an anesth etic mixture of medetomidine (0.3 mg/kg, Medetomin injection Meiji; Meiji Seika Pharma), midazolam (4.0 mg/kg, Midazolam Injection Sandoz; Sandoz K. K), and butorphanol tartrate (5.0 mg/kg, Vetorphale; Meiji Seika Pharma).

### 2.4 Fertility test

To evaluate male fertility, a single 12-week-old male mouse (control or *Kif2c* cKO) was caged with two 8-week-old female Wild Type (WT) mice for 2 months. Mating was verified by monitoring for the presence of vaginal plugs. Plugged females were replaced with fresh mice. Litter size was scored for females with plugs. To evaluate female fertility, two 8-week-old female mice (control and *Kif2c* cKO) were caged with a 12-week-old male WT mouse for 5 months. Female mice were separated their pups immediately after the litters and were mated again 1 week later. This process was repeated five times, and the cumulative litter size was scored.

### 2.5 RT-qPCR

RNA extraction was performed using NucleoSpin® RNA (U0955S, TaKaRa Bio) following the manufacturer’s protocol. Briefly, tissues were homogenized in buffer RA1, after which the filtrate solution was incubated DNase reaction mixture and washed buffer RAW2 and RAW3 three times. The washed solution was extracted with RNase-free H_2_O via centrifugation. Reverse transcription was conducted using ReverTra Ace -α-® (FSK-101F, TOYOBO) following the manufacturer’s protocol to obtain cDNA. The obtained cDNA was amplified through qPCR using TB Green® Premix Ex Taq™ II (RR820S; TaKaRa Bio). The primer sequences used were as follows: Fw: 5′- ATGGAGTCGCTTCACGCAC-3′. Rv: 5′- CCACCGAAACACAGGATTTCTC-3′.

### 2.6 Histological analysis

The testes and ovaries were collected from control mice and *Kif2c* cKO mice, and testis weight were measured. Testes were fixed in Bouin’s solution, and ovaries were fixed in 4% formaldehyde. The fixed tissues were gradually dehydrated using a graded series of ethanol and xylene solutions at different concentrations and finally embedded in paraffin. Sections were rehydrated, testes were stained with periodic acid–Schiff (PAS), and ovaries were stained with hematoxylin and eosin and examined under a microscope (BX50; Olympus).

### 2.7 Immunohistochemistry

Immunohistochemistry (IHC) was performed as previously described (Hiradate et al., 2022). Tissue sections were placed in an antigen retrieval buffer (pH 9.0; 1 × TE buffer) for 20 min at 90°C. The following primary antibodies were used: rabbit anti-KIF2C antibody (PA5-109879, Thermo Fisher Scientific), mouse anti-SCP3 antibody (sc-74569, Santa Cruz), mouse anti-α-tubulin antibody (sc-32293, Santa Cruz), mouse anti-DDX4/MVH antibody (ab27591, Abcam). The acrosomes were stained with Alexa Fluor 568–conjugated Lectin PNA (L32458; Thermo Fisher Scientific). Sections were examined under a fluorescence microscope (BZ-X710; Keyence).

### 2.8 TdT-mediated dUTP nick-end labeling (TUNEL) assay

The TUNEL assay was performed using an *in situ* apoptosis detection kit (MK500, TaKaRa Bio) as previously described (Hiradate et al., 2022). Briefly, paraffin-embedded testes sections at 8 weeks were rehydrated and labeled with FITC-dUTP by TdT enzyme for 75 min at 37°C. The sections were washed thrice with PBS and incubated with Hoechst 33,342 for nuclear staining. The sections were examined under a fluorescence microscope (BZ-X710, Keyence). One hundred randomly chosen seminiferous tubules per mouse were examined, and TUNEL signals that merged with the nucleus were counted as TUNEL-positive.

### 2.9 Immunocytochemistry of testicular germ cells

To stain individual cells, samples were prepared using the following protocol: seminiferous tubules were fixed in 2% formaldehyde containing 0.1% Triton X-100 for 10 min at room temperature (RT). Pieces of tubules were placed in a drop of fixing solution on a glass slide and the pieces were minced in the drop. The slides were gently tapped onto coverslips using tweezers, frozen in liquid nitrogen for 30 s, and then the coverslips were removed. The slides were washed three times with PBS for 5 min. The slides were treated with blocking buffer (Blocking One, 03953-95, Nacalai Tesque) for 1 h at RT and incubated with the primary antibody overnight at 4°C. The following primary antibodies were used: mouse anti-α-tubulin (Santa Cruz), rabbit anti-α-tubulin (ab52866, Abcam), mouse anti-γ-tubulin (T6557, Sigma-Aldrich), rabbit anti-KIF2C (Thermo Fisher Scientific), rabbit anti-BubR1 (11504-2-AP, Proteintech), and mouse anti-SCP3 (Santa Cruz). The slides were washed thrice with PBS containing 0.1% Tween 20 for 5 min and incubated with Alexa Fluor-dye secondary antibodies (Thermo Fisher Scientific) for 1 h at RT. The ovaries were examined under a fluorescence microscope (BZ-X710; Keyence).

### 2.10 Ovarian follicles count

Paraffin-embedded ovaries at 8 weeks were sectioned at 5 µm and the ovarian follicles were counted from every fifth serial section, with 20 sections per ovary. The follicles were divided into primordial (POF), primary (PF), secondary (SF), and antral (AF) follicles and counted without duplication.

### 2.11 Immunocytochemistry of oocytes

To collect oocyte at metaphase Ⅱ, 8-weeks-old mice were superovulated by intraperitoneal injection of 5 IU pregnant mare serum gonadotropin (SEROTROPIN^Ⓡ^, Asuka Animal Health) and after 48 h, intraperitoneally injected 5 IU human chorionic gonadotropin (HCG; Mochida Pharmaceutical). Fifteen hours after hCG injection, cumulus-oocyte complexes were collected, and cumulus cells were removed in HTF medium containing 0.1% hyaluronidase (18240-36, Nacalai Tesque) for 5 min at 37.5°C with 5% CO_2_. The composition of HTF medium is follow: 101.6 mM NaCl, 4.7 mM KCl, 0.37 mM K_2_PO_4_, 0.2 mM MgSO_4_·7H _2_ O, 2 mM CaCl_2_, 25 mM NaHCO_3_, 2.78 mM glucose, 0.33 mM sodium pyruvate, 21.4 mM sodium lactate, 286 mg/L penicillin G, and 228 mg/L streptomycin. Oocytes were washed three times with washing buffer (0.1% polyvinyl alcohol and 1% Bovine Serum Albumin in PBS) and fixed with 2% formaldehyde containing 0.2% Triton X-100 in PBS for 40 min at RT. Oocytes were washed thrice with washing buffer and treated with blocking buffer (Blocking One) for 1 h at RT. Oocytes were incubated with the primary antibody overnight at 4°C. The following primary antibodies were used: mouse anti-α-tubulin (Santa Cruz), rabbit anti-α-tubulin (ab52866, Abcam), and rabbit anti-KIF2C (Thermo Fisher Scientific). The oocytes were washed thrice with washing buffer for 5 min and incubated with Alexa Fluor-dye secondary antibodies (Thermo Fisher Scientific) for 1 h at RT. Samples were examined under a fluorescence microscope (BZ-X710, Keyence).

### 2.12 Western blotting

Testes were lysed using RIPA buffer (08714-04, Nacalai Tesque) containing 1% phosphatase inhibitor (07575-51, Nacalai Tesque) and incubated for 30 min on ice. The lysates were centrifuged at 15,000 × g for 15 min at 4°C and eluted with the sample buffer for 5 min at 100°C. Proteins were separated by SDS-PAGE, transferred to a PVDF membrane, and treated with a blocking buffer (Blocking One, 03953-95, Nacalai Tesque) for 30 min at RT. Membranes were then incubated with the primary antibody overnight at 4°C. The following primary antibodies were used: rabbit anti-KIF2C (Thermo Fisher Scientific) and mouse anti-β-actin (sc-47778, Santa Cruz). After washing three times with TBS-T for 5 min, the membranes were incubated with anti-rabbit or anti-mouse HRP-conjugated antibodies (W4011 and W4021, Promega) for 1 h at RT. Images were captured using an ImageQuant LAS 500 (Cytiva).

### 2.13 Statistical analyses

Each experiment was conducted in at least three independent mice of each genotype. The quantitative analyses were used BZ-X analyzed (BZ-X710, Keyence). Statistical tests were used KyPlot 6.0 version 6.0.2 (KyensLab Inc.). Sample sizes, statistical tests, and *p*-values are showed in the manuscript, figures, and figure legends.

## 3 Results

### 3.1 KIF2C localizes to centromeres at meiotic metaphase and to the manchette in elongated spermatids

**FIGURE 1:**
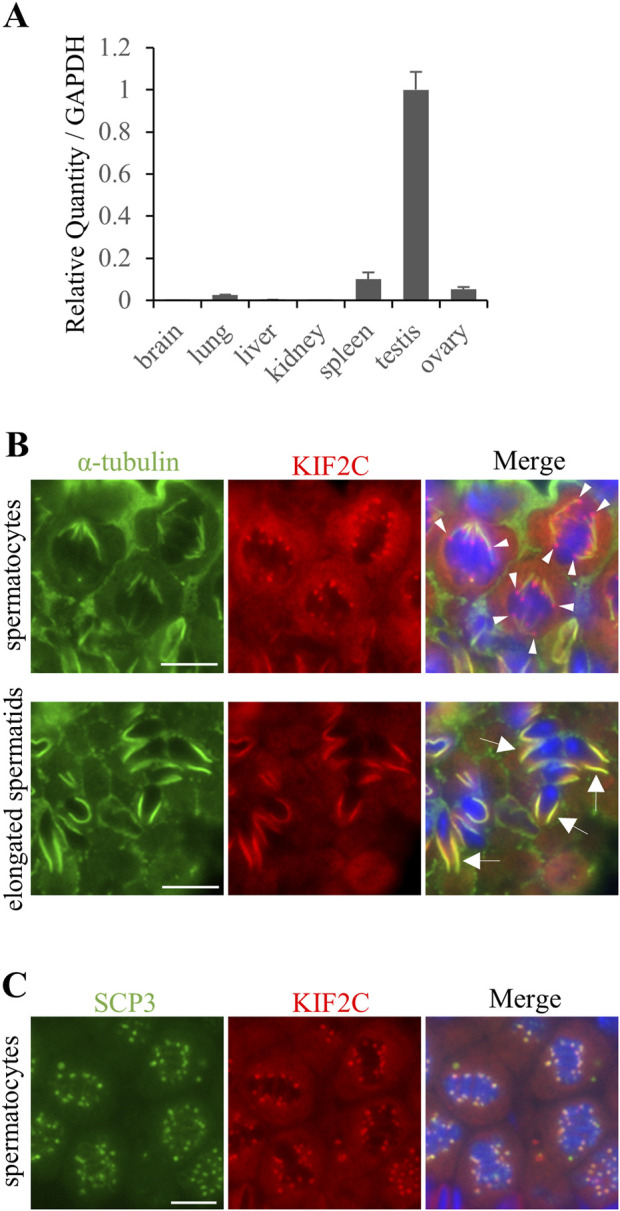
KIF2C was localized to centromeres at meiosis metaphase and along the manchette in elongated spermatids. **(A)** mRNA expression of *Kif2c* using RT-qPCR at 8-week-old WT mice. Error bars indicate means ± S.D. **(B)** The localization analysis of KIF2C in 8-week-old WT mice testes using IHC (green: anti-α-tubulin, red: anti-KIF2C, blue: Hoechst 33,342). Arrowheads indicate punctate signals on chromosomes of KIF2C. Arrows indicate the signals along the manchette in elongated spermatids. Scale bars = 10 µm. **(C)** The localization analysis of KIF2C at meiotic metaphase in spermatocyte in 8-week-old WT mice using IHC (green: anti-SCP3, red: anti-KIF2C, blue: Hoechst 33,342). Scale bars = 10 µm.

RT-qPCR analysis of various tissues showed that *Kif2c* mRNA was highly expressed in the adult testis (Figure 1A). Next, we performed immunohistochemistry to determine KIF2C localization in germ cells of the testis. We found that KIF2C localized as a punctate on chromosomes at the spindle tip at metaphase in spermatocytes (Figure 1B arrowheads at top). Moreover, KIF2C localized along the manchette, a microtubule-based skirt-like transient structure around the nucleus of elongating spermatids (Figure 1B arrows at bottom). SCP3 accumulates in centromeres at metaphase in meiosis (Parra et al., 2009; Parra et al., 2006). Therefore, we examined the definite localization of KIF2C by co-staining with SCP3. KIF2C and SCP3 colocalized at metaphase in meiosis (Figure 1C). Therefore, KIF2C localized to centromeres at metaphase in meiosis. In contrast, KIF2C did not localize to the spindle tip at mitotic metaphase in spermatogonia (Supplementary Figure S1A). These results suggest that KIF2C functions at centromeres in meiotic metaphase spermatocytes and at the manchette in elongated spermatids.

### 3.2 Germ cell-specific *Kif2c* knockout mice causes male infertility

**FIGURE 2:**
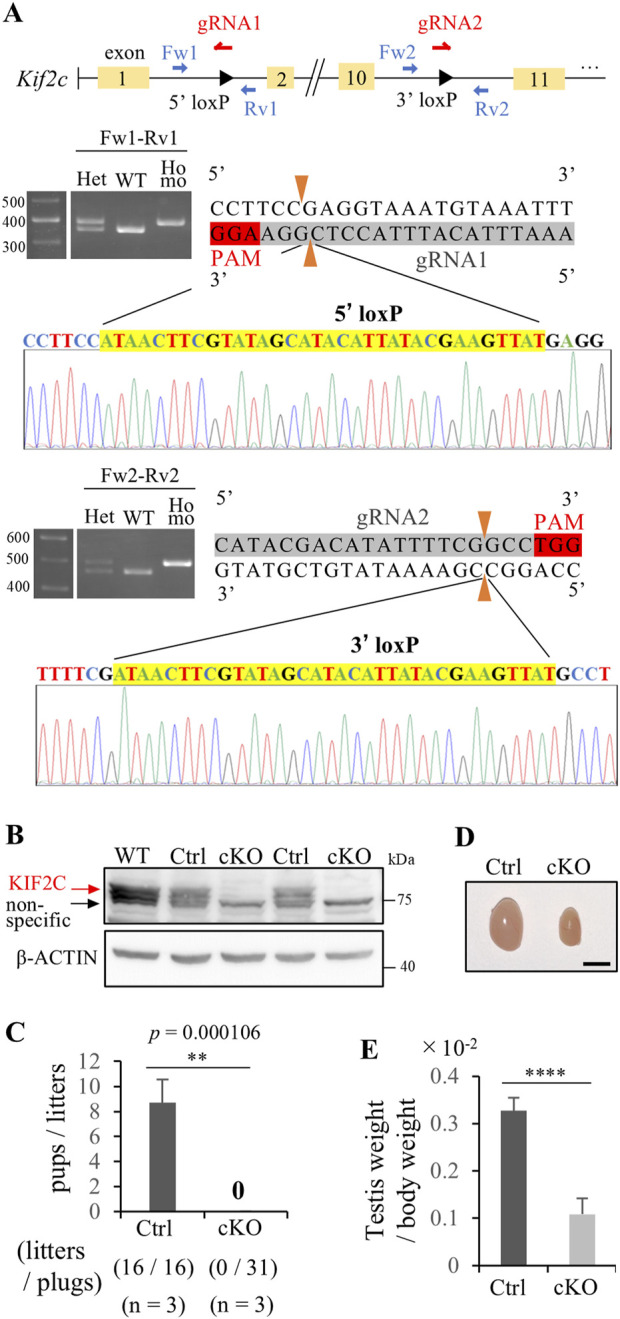
*Kif2c* cKO mice showed male infertility. **(A)** Schematic representation of the generation of *Kif2c* ^flox/flox^ mice. loxP sites were inserted flanking between exon 2 and 10. loxP sites insertion at target location was confirmed using Genotyping and Sanger sequencing. **(B)** WB analysis in WT, Ctrl and *Kif2c* cKO mice. β-ACTIN was used as loading control. **(C)** Fertility test of Ctrl and *Kif2c* cKO mice (n = 3 for Ctrl mice and *Kif2c* cKO mice). We observed 16 plugs in Ctrl mice and 31 plugs in *Kif2c* cKO mice. Error bars indicate means ± S.D. **(D)** The testis of Ctrl and *Kif2c* cKO mice at 12 weeks. Scale bars = 5 mm. **(E)** The ratio of testes weight/body weight of Ctrl and *Kif2c* cKO mice at 12 weeks (n = 3 for Ctrl and *Kif2c* cKO mice). Error bars indicate means ± S.D. All analysis is used two-tailed Student’s t-test. *p < 0.05, **p < 0.01, ****p < 0.0001.

We created germ cell-specific *Kif2c* conditional knockout mice using the Cre-loxP system. We designed a pair of gRNA and ssODN to insert loxP sites flanking exons 2 and 10 using *i*-GONAD methods (Gurumurthy et al., 2019) (Figure 2A). We confirmed the successful incorporation of the loxP 34 bp sequence at the target site through genotyping and Sanger sequencing (Figure 2A). Subsequently, the generated *Kif2c* ^flox/flox^ mice (loxP sites were inserted homozygously into the *Kif2c* gene) were crossed with Ddx4-Cre (Vasa-Cre) transgenic mice to generate germ cell-specific *Kif2c* homozygous knockout (*Kif2c* ^flox/-^; Vasa-cre: *Kif2c* cKO) mice. *Kif2c* ^flox/+^; Vasa-cre mice and *Kif2c* ^+/−^; Vasa-cre mice are heterozygous knockout mice in germ cells; we used *Kif2c* ^flox/+^; Vasa-cre mice as control (Ctrl) mice for male analysis and *Kif2c* ^+/−^; Vasa-cre mice as Ctrl mice for female analysis. Both Ctrl mice showed normal fertility, comparable to that of WT mice (data not shown). Western blot (WB) analysis confirmed that KIF2C proteins were almost completely lost in *Kif2c* cKO mouse testes (Figure 2B). Furthermore, IHC confirmed that the expression of KIF2C disappeared in the male germ cells of *Kif2c* cKO mice (Supplementary Figure S1B arrowheads and arrows). Next, we assessed the fertility of *Kif2c* cKO male mice and found that *Kif2c* cKO mice were completely infertile (Figure 2C). Furthermore, the testis size was also significantly smaller (Figure 2D), and the testis weight of the *Kif2c* cKO mice was significantly lower than that of the Ctrl mice (Figure 2E). These results indicate that *Kif2c* is essential for male fertility.

### 3.3 *Kif2c* deletion in male germ cells causes the abnormal chromosomes congression at meiotic metaphase and induces apoptosis

**FIGURE 3:**
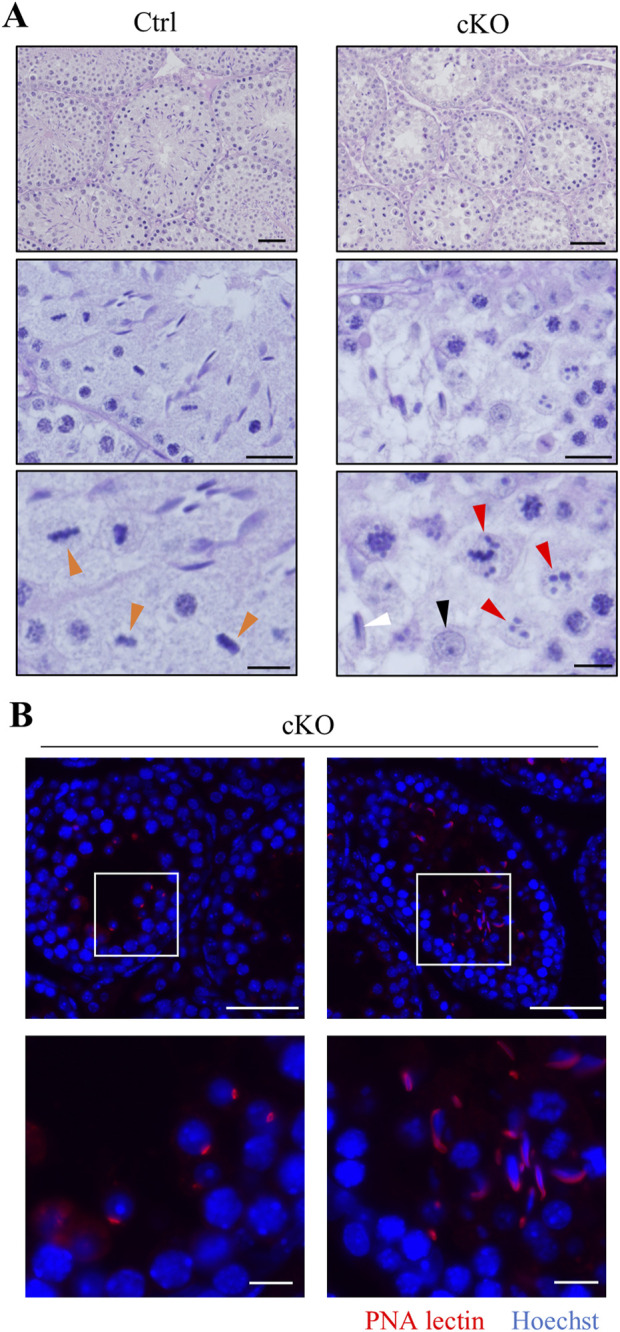
*Kif2c* cKO male mice cause abnormal chromosome congression, and induce germ cell reduction. **(A)** PAS-H staining of seminiferous tubules in Ctrl and *Kif2c* cKO mice at 8 weeks. the low panels were high magnification. Scale bars = 50 µm (top panels), 20 µm (middle panels), 10 µm (bottom panels). Orange arrowheads indicate normal chromosome congression, red arrowheads indicate abnormal chromosome congression, the black is round spermatid, and the white is elongated spermatid. **(B)** The acrosome stain to detect round spermatids and elongated spermatids. (red: PNA lectin, blue: Hoechst 33,342). The white boxes are high magnification areas. Scale bars = 50 µm (low magnification) and 10 µm (high magnification).

**FIGURE 4:**
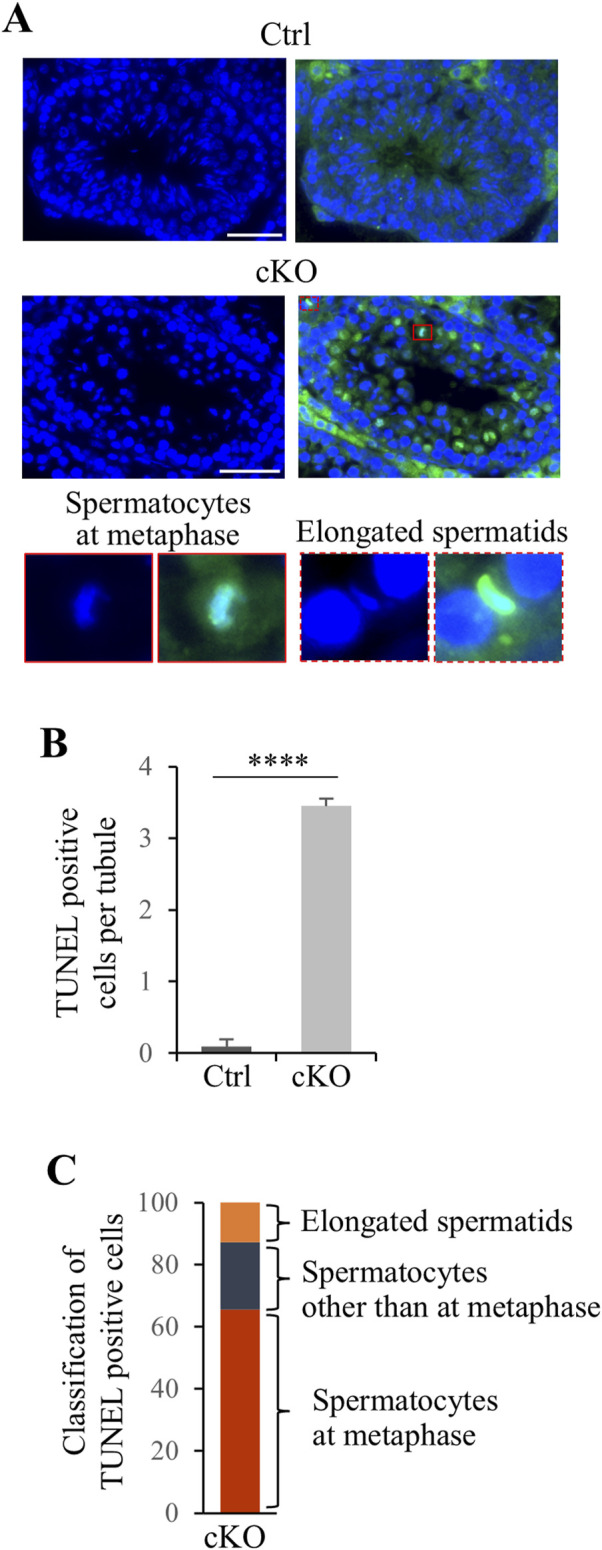
Apoptosis cell were significant increase in *Kif2c* cKO male mice. **(A)** TUNEL assay in Ctrl and *Kif2c* cKO mice at 8 weeks. The red box is TUNEL positive cell in metaphase spermatocyte and the red dashed box is elongated spermatid. Scale bars = 50 µm. **(B)** The ratio of TUNEL positive cells per tubules in Ctrl and *Kif2c* cKO mice. 100 tubules per mouse were examine. Error bars indicate means ± S.D. Two-tailed Student’s t-test, n = 3, *p < 0.05, **p < 0.01, ****p < 0.0001. **(C)** Classification of TUNEL positive cells in *Kif2c* cKO mice.

To investigate the cause of male infertility and decreased testicular weight in *Kif2c* cKO mice, we performed a histological analysis of seminiferous tubules using periodic acid-Schiff hematoxylin (PAS-H) staining. Only a few haploid germ cells were present in *Kif2c* cKO mice compared to Ctrl mice (Figure 3A, low magnification). In more detailed observation, chromosomes aligned at the equator in meiotic metaphase spermatocyte in Ctrl mice (Figure 3A, orange arrowheads at high magnification). However, chromosome misalignments at meiotic metaphase were detected in the majority of spermatocytes in *Kif2c* cKO mice (Figure 3A, red arrowheads at high magnification). Round and elongated spermatids were not completely absent and only a few spermatids were present (Figure 3A; black and white arrowheads at high magnification; Figure 3B). These results suggest that the number of haploid germ cells was significantly decreased due to chromosomal misalignments at meiotic metaphase in *Kif2c* cKO mice. To investigate the cause of germ cell decrease, we performed a TdT-mediated dUTP nick-end labeling (TUNEL) assay. TUNEL assay showed that the number of apoptotic cells was significantly increased in *Kif2c* cKO mice (Figure 4A). Many TUNEL-positive cells were spermatocytes at metaphase, and apoptotic signals were often detected in elongated spermatids (Figures 4B, C). These results indicate that cell death was frequently occurred at metaphase spermatocyte in *Kif2c* cKO mice. A few spermatocytes differentiated into spermatids in *Kif2c* cKO mice, however cell death was also induced in elongated spermatids in *Kif2c* cKO mice. Furthermore, no spermatozoa are present in cauda epididymis in *Kif2c* cKO mice (Supplementary Figure S2). These results suggest that some kind of abnormality inducing cell death occurred in elongated spermatids in *Kif2c* cKO mice.

### 3.4 Chromosomes alignment at metaphase in spermatocyte and manchette dynamics in elongated spermatids were impaired in *Kif2c* cKO mice

**FIGURE 5:**
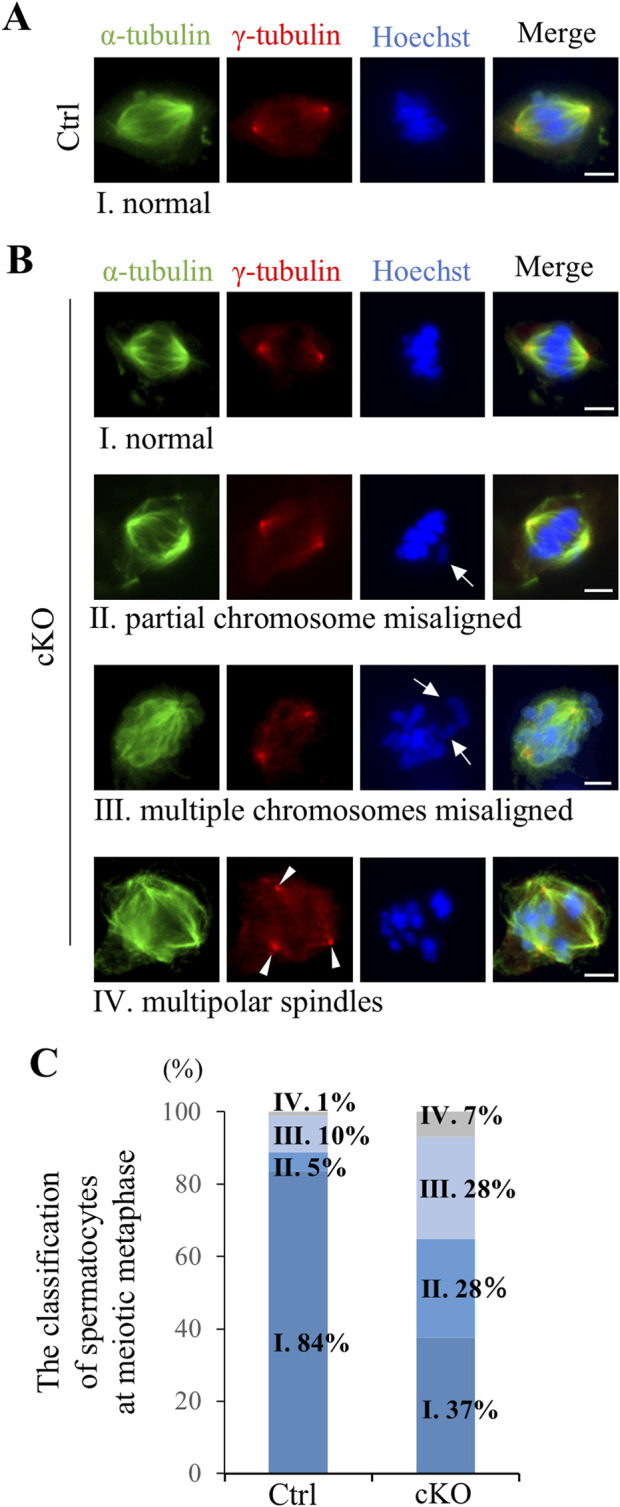
*Kif2c* cKO male mice showed chromosomes misalignment at meiotic metaphase. **(A, B)** The analysis of spindle structure and chromosomes alignment at PD20-21 spermatocyte using ICC (green: anti-α-tubulin, red: anti-γ-tubulin, blue: Hoechst 33,342). Ⅰ-Ⅳ indicate spermatocytes at meiotic metaphase observed in Ctrl and *Kif2c* cKO mice. Ⅰ: Normal chromosome alignment, Ⅱ: partial chromosome misalignment, Ⅲ: multiple chromosomes misalignment, Ⅳ: multipolar spindles. Arrows indicate misaligned chromosomes and arrowheads indicate multipolar spindle. Scale bars = 10 µm. **(C)** The ratio of the defects observed meiotic metaphase in Ctrl and *Kif2c* cKO mice (n = 180 cells for Ctrl mice and n = 173 cells for *Kif2c* cKO mice). Spermatocytes at metaphase were collected from three different mice for Ctrl and *Kif2c* cKO mice, respectably.

**FIGURE 6:**
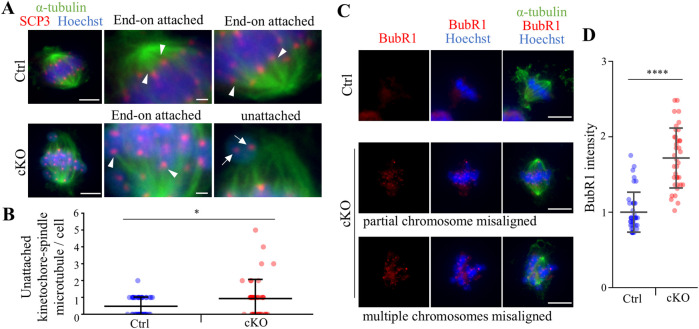
*Kif2c* cKO male mice showed some abnormalities at meiotic metaphase. **(A)** The analysis of kinetochore - spindle attachment at PD20-21 spermatocyte using ICC (green: anti-α-tubulin, red: anti-SCP3, blue: Hoechst 33,342). Arrowheads indicate End-on attachment between kinetochore and spindle, and arrows indicate unattachment. Scale bars = 5 µm (low magnification) and 1 µm (high magnification). **(B)** The quantification of unattached kinetochore-spindle microtubule per cell (n = 46 cells for Ctrl mice and n = 43 cells for *Kif2c* cKO mice). Error bars indicate means ± S.D. **(C)** The analysis of SAC PD20-21 spermatocyte using ICC (green: anti-α-tubulin, red: anti-BubR1, blue: Hoechst 33,342). Scale bars = 10 µm. **(D)** The quantification of BubR1 intensity in Hoechst (n = 39 cells for Ctrl mice and n = 37 cells for *Kif2c* cKO mice). The intensity was normalized by the average value of Ctrl intensity. Error bars indicate means ± S.D. All analysis is used two-tailed Weich’s t-test. *p < 0.05, **p < 0.01, ****p < 0.0001.

To analyze defects in spermatocyte metaphase in *Kif2c* cKO mice, we conducted immunocytochemistry on spermatocytes from postnatal day (PD) 20–21 testis, and examined meiotic spindle structure and chromosome alignment. In *Kif2c* cKO mice, three main types of defects were more frequently observed than in Ctrl mice (Figures 5A–C). The first was partial chromosomal misalignment (Figure 5BII), the second was multiple misaligned chromosomes (Figure 5BIII), and the third was multipolar spindles (Figure 5BIV). The majority of defects observed in metaphase spermatocytes of *Kif2c* cKO mice were chromosomes misalignment (Figure 5C). In addition, 37% of spermatocytes were showed normal chromosome alignment (Figure 5C). Therefore, chromosome congression at the equator were impaired in *Kif2c* cKO mice. Next, we investigated the attachment between the kinetochores and the spindle microtubules in *Kif2c* cKO mice. Unattached kinetochore-spindle microtubule was increased in *Kif2c* cKO mice spermatocytes, but many cells with normal attached were observed in Kif2c cKO mice (Figures 6A, B). Kinetochore-spindle microtubule attachment and chromosome alignment at the equator are monitored and regulated by a ubiquitous safety device called the spindle assembly checkpoint (SAC) (Burke and Stukenberg, 2008; Caudron et al., 2005; Cleveland et al., 2003; Musacchio and Salmon, 2007; Nasmyth, 2001). When the spindle microtubules have correctly attached to kinetochores and aligned at the equator, SAC is inhibited and it proceeds to anaphase (Musacchio and Salmon, 2007). Thus, we analyzed the behavior of SAC system. The SAC component protein BubR1 was accumulated in metaphase spermatocytes of *Kif2c* cKO mice (Figures 6C, D). In *Kif2c* cKO mice, SAC is activated due to chromosome misalignment, leading to meiotic arrest at metaphase. These results suggest that KIF2C functions in the regulation of kinetochore microtubule flux to align chromosomes, rather than in spindle formation or kinetochore-spindle microtubule attachment.

**FIGURE 7:**
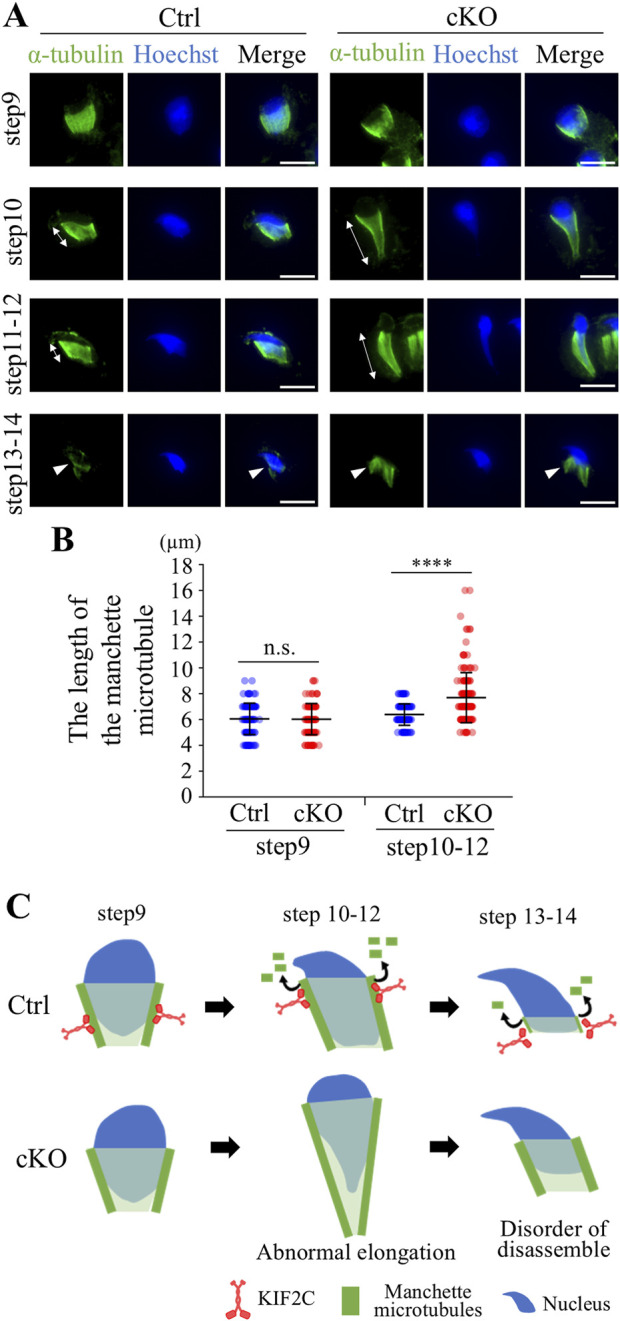
*Kif2c* cKO mice showed impairment of the manchette dynamics. **(A)** The analysis of the manchette structure in step9-14 elongated spermatids at 8-week-old using ICC (green: anti-α-tubulin, blue: Hoechst 33,342). White double-headed arrows indicate the length between the perinuclear ring and caudal side of the nucleus. Arrowheads indicate the manchette microtubules during disassemble process. Scale bars = 10 µm. **(B)** The quantification of the length of the manchette microtubules indicated by white double-headed arrows (Ctrl mice: n = 141 cells for step9 and n = 167 for step10-12. *Kif2c* cKO mice: n = 134 cells for step9 and n = 169 cell for step10-12.). Error bars indicate means ± S.D. Two-tailed Welch’s t-test. n.s.: no significant. *p < 0.05, **p < 0.01, ****p < 0.0001. **(C)** Schematic diagram of the manchette abnormalities in *Kif2c* cKO mice. KIF2C regulates manchette dynamics by depolymerizing manchette microtubules.

Because KIF2C localized to the manchette in elongated spermatids, KIF2C may also function in the regulation of microtubule dynamics in the manchette. We analyzed the structure of the manchette through staining for α-tubulin using ICC. The manchette begins to appear in step 9 of elongated spermatids, gradually moves to the caudal end of the head, and disassembles in step 14 (Figure 7A Ctrl). In *Kif2c* cKO mice, the manchette is normally formed and maintained in step 9 elongated spermatids compared to that in *Ctrl* mice (Figure 7A). However, in step 10-12 elongated spermatids, the head structure was abnormally elongated, and the manchette showed abnormal morphologies, such as being overly elongated in *Kif2c* cKO mice (Figures 7A, B). Moreover, in step 13-14 elongated spermatids, which occurred when the manchette was gradually disassembled, were not disassembled in *Kif2c* cKO mice (Figure 7A). These results show that the manchette dynamics were compromised in *Kif2c* cKO mice (Figure 7C). Therefore, it is suggested that KIF2C regulates microtubule dynamics in the manchette by depolymerizing microtubules and maintains manchette structure (Figure 7C).

### 3.5 KIF2C is not essential for female fertility

**FIGURE 8:**
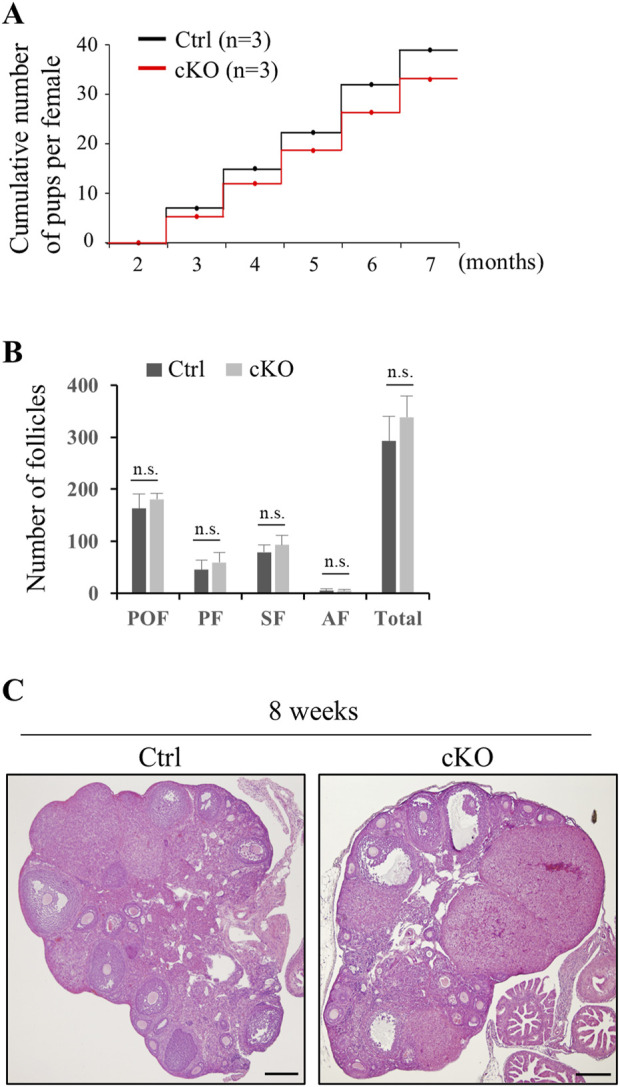
*Kif2c* cKO female mice were sufficiently fertile. **(A)** Fertility test of *Kif2c* cKO female mice (n = 3 for Ctrl and *Kif2c* cKO mice). Cumulative number of pups per female was counted by five litters over 5 months. **(B)** Number of follicles at 8-week-old Ctrl and *Kif2c* cKO mice. Error bars indicate means ± S.D. Two-tailed Student’s t-test, n = 3, n.s.: no significant. **(C)** HE staining of ovary in Ctrl and *Kif2c* cKO mice at 8 weeks. Scale bars = 200 µm.

**FIGURE 9:**
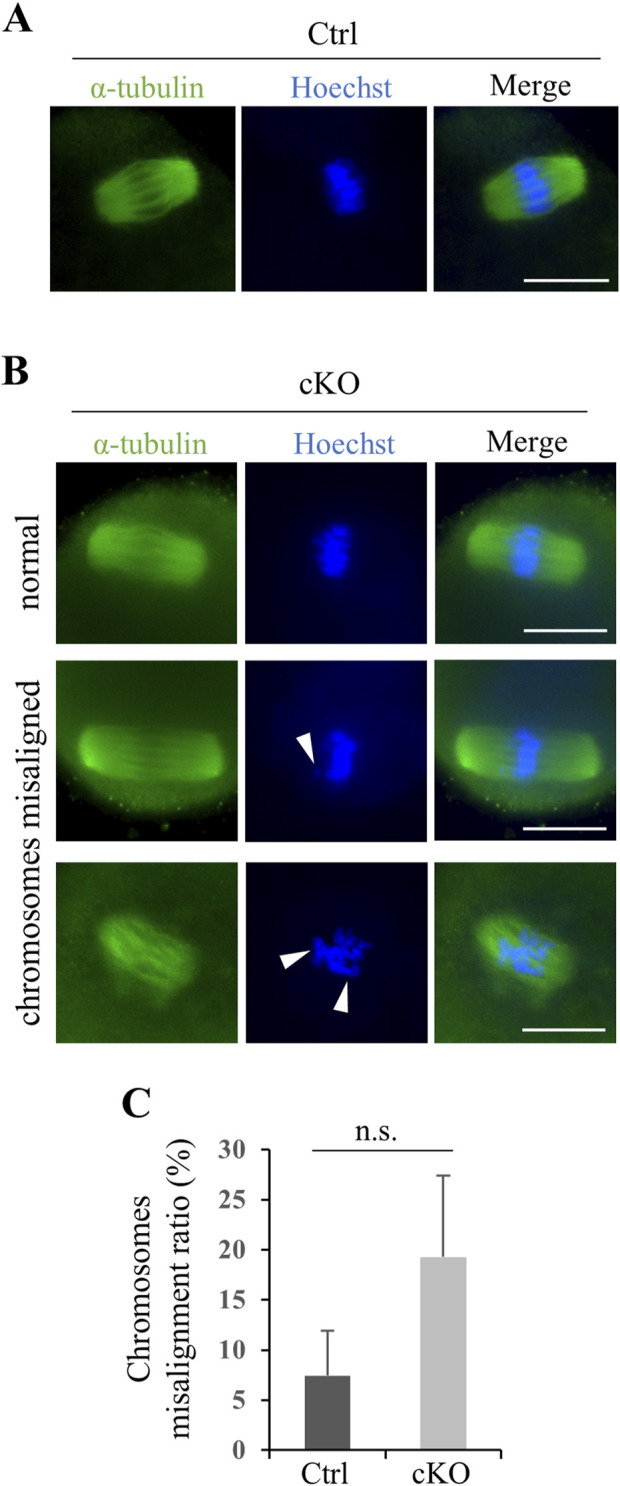
*Kif2c* cKO female mice had only a slight effect on chromosomes alignment in oocyte meiosis. **(A, B)** The analysis of spindle structure and chromosomes alignment at 8-week-old oocytes using ICC (green: anti-α-tubulin, Hoechst 33,342). Arrowheads indicate misaligned chromosomes. Scale bars = 20 µm. **(C)** The ratio of chromosomes misalignment in oocyte (n = 100 cells for Ctrl mice and n = 93 cells for *Kif2c* cKO mice). Oocytes at metaphase Ⅱ were collected from three different mice for Ctrl and *Kif2c* cKO mice, respectably. Error bars indicate means ± S.D. Two-tailed Student’s t-test, n = 3, n.s.: no significant.

In oocyte, KIF2C localize to centromeres in meiotic metaphase (Illingworth et al., 2010; Vogt et al., 2010), and thus we investigated the effect of KIF2C deficit in female fertility and chromosome alignment in oocyte. In contrast to male mice, female mice had sufficient fertility, and even after repeated litters, fertility did not decline and was maintained (Figure 8A). The number of follicles in *Kif2c* cKO mice was comparable to that in the Ctrl mice (Figure 8B), and the morphology of the ovaries was also normal (Figure 8C). As chromosome misalignment is frequently observed in metaphase spermatocytes, we analyzed chromosome congression in metaphase oocytes using ICC. Chromosome misalignment was slightly increased in *Kif2c* cKO mice, but there was no significant difference compared with that in Ctrl mice (Figures 9A–C). These results showed that unlike *Kif2c* cKO male mice, *Kif2c* cKO female mice had sufficient fertility for a long period, and chromosome alignment in the meiotic metaphase was minorly affected.

## 4 Discussion

In the present study, we found that KIF2C plays an important role in chromosomes alignment in male meiosis and in manchette dynamics in elongated spermatids. KIF2C inhibition or depletion in mitosis leads to chromosome misalignment, segregation errors, and lagging chromosomes in anaphase, owing to a reduction in chromosome velocity (Bakhoum et al., 2009; Ganem et al., 2005; Kline-Smith et al., 2004; Maney et al., 1998), however no serious abnormalities, such as mitotic arrest or cell death, are observed (Kline-Smith et al., 2004). In this study, *Kif2c* cKO male mice caused a severe phenotype with meiotic arrest and cell death in spermatocytes. Therefore, there are differences in the functions of KIF2C in chromosomes alignment between mitosis and male meiosis. In contrast to male, *Kif2c* cKO female mice had sufficient fertility, and the proportion of oocytes showing chromosomal misalignment in the meiotic metaphase was much lower than that in males. These results are supported by a previous study, which showed that the proportion of chromosome misalignment is approximately 30% when KIF2C is depleted or inhibited (Illingworth et al., 2010). Furthermore, even among oocytes that showed chromosomal abnormalities due to KIF2C inhibition, many complete meiosis normally (Illingworth et al., 2010), suggesting that KIF2C is not essential for meiosis progression in oocytes. Therefore, KIF2C may be play a critical role in male meiosis, rather than in mitosis or female meiosis. The different phenotypes of males and females in *Kif2c* cKO mice are intriguing. In previous studies, KIF2A, which belongs to the kinesin 13 family same as KIF2C, plays an important role in chromosomes alignment in oocyte meiosis (Chen et al., 2016; Yi et al., 2016). KIF2A depletion in oocytes causes severe spindle defects and chromosomal misalignment, and the chromosome misalignment ratio is 50%–80% (Chen et al., 2016; Yi et al., 2016), which is much higher than KIF2C depletion oocytes in this study and a previous study (Illingworth et al., 2010). In addition, by KIF2A depletion, SAC is activated in oocytes with chromosomal misalignment, and most of these oocytes are arrested at metaphase I (Chen et al., 2016; Yi et al., 2016). These studies show that KIF2A deficiency results in a more severe phenotype than KIF2C deficiency in oocyte meiosis. Therefore, KIF2A may function more preferentially than KIF2C during oocyte meiosis, or it is possible that the compensation by KIF2A works in *Kif2c* cKO female mice. In males, the involvement of KIF2A in meiosis is unclear; however, KIF2A is known to localize to the manchette and flagella (Hu et al., 2023; Krähling et al., 2013). Therefore, it is thought to KIF2A functions mainly in the manchette and flagellum during spermatogenesis. On the other hand, another explanation in male specific phenotype in *Kif2c* cKO mice may be the differences in microtubule dynamics between male and female germ cells. Spindle formation during meiosis differs between males and females (Gatti et al., 2012; Kallio et al., 1998). In males, the spindle is formed from the two centrosome poles in mitosis, whereas in females, there are no canonical centrosome, which is defined as two structures of centriole and pericentriolar material surrounded it (Bettencourt-Dias et al., 2011), and the spindle is formed near chromosome (Heald et al., 1996). In addition, the dynamics of microtubule organizing center is unique in female meiosis. In mice, microtubule organizing center, centriole, is dissolved in germinal vesicle-stage prior to microtubule assembly and separated centrioles lose its function as microtubule organizing center, but these non-functional centrioles present through meiotic metaphase Ⅰ and Ⅱ (Simerly et al., 2018). Therefore, further study is needed to determine whether male specific phenotype in *Kif2c* cKO mice reflects the differences in the function of KIF2C between male and female in meiosis. The manchette transiently assembles in elongated spermatids; however, the regulatory mechanism, from its appearance to its removal, is largely unknown. In *Kif2c* cKO male mice, the manchette showed abnormal morphology and delayed disassembly in elongated spermatids, suggesting that KIF2C regulates the manchette dynamics. In previous study, the protein expression of KIF2C was reduced in the manchette of *Camsap1* ^−/−^ mice in which manchette disassembly was delayed (Hu et al., 2023). Thus, KIF2C functions in microtubule depolymerization in the manchette. In *Kif2c* cKO male mice, the disorder of microtubule depolymerization leads to impaired microtubule dynamics in the manchette and abnormal manchette morphology. CAMSAP1 localize at the minus end of the manchette (Hu et al., 2023), however KIF2C localized along the entire manchette and the localization pattern is not completely consistent. Moreover, KIF2C only localized to centromeres, which is the plus end of spindle microtubule, at meiotic metaphase. Therefore, KIF2C may preferentially localize at the microtubule plus end in male germ cells and be responsible for plus end depolymerization in the manchette. When manchette dynamics are compromised in elongated spermatids, differentiation of elongated spermatids is arrested (Lehti and Sironen, 2016). In *Kif2c* cKO male mice, 37% of spermatocytes showed normal chromosome alignment and elongated spermatids were present in testis. Therefore, spermatocytes complete meiosis normally at a certain ratio in *Kif2c* cKO mice. However, subsequent differentiation arrest occurs in elongated spermatids owing to abnormal manchette dynamics. Indeed, apoptosis was detected in many elongated spermatids in the present study. Therefore, severe disorders occur in both meiosis and manchette in *Kif2c* cKO male mice, which is the main cause of male infertility in *Kif2c* cKO mice.

*Kif2*c mRNA is highly expressed in human testis as well mouse (Uhlén et al., 2015), and the expression pattern of *Kif2c* mRNA is similar between mouse and human by single-cell RNA sequencing in male germ cells (Green et al., 2018). In addition, downregulation of KIF2C is associated with azoospermia (Cao et al., 2021). Therefore, it is thought that KIF2C is important for microtubule dynamics in human spermatogenesis. However, the mechanism of microtubule dynamics of meiosis and manchette in human spermatogenesis is largely unknown, and there may be some different mechanism between human and mouse. Therefore, further studies are needed whether the function of KIF2C in mouse spermatogenesis can be directly reflected in human. In addition, the expression of *Kif2c* gene is significantly reduced in the testes of patients with Klinefelter syndrome (KS) according to recent study (He et al., 2022). KS is accompanied by severe spermatogenesis disorders including non-obstructive azoospermia (Aksglaede et al., 2006). Since KS greatly affect the gene expression in the testis (Winge et al., 2018a; Winge et al., 2018b), the recent studies have focused on the regulation of gene expression to elucidate the cause of spermatogenesis disorders in KS. Therefore, the findings of KIF2C function in spermatogenesis revealing in this study may help to clear the mechanism of spermatogenesis disorders and to target new treatment methods in KS.

In summary, we identified *Kif2c* as a novel gene that is essential for male fertility. KIF2C controls chromosomes alignment at meiotic metaphase and manchette dynamics in elongated spermatids by depolymerizing the microtubules. In contrast, KIF2C is not essential for female fertility, and the loss of KIF2C only slightly affected chromosomes alignment in oocytes. In this study, we demonstrate that KIF2C plays important role in microtubule dynamics in many processes of spermatogenesis. These findings provide novel insights into the molecular mechanisms underlying unique microtubule dynamics of spermatogenesis.

## Data Availability

The original contributions presented in the study are included in the article/Supplementary Material, further inquiries can be directed to the corresponding author.
