# Supplementary material for: KIF2C is essential for meiosis and manchette dynamics in male mice
Source: Front Cell Dev Biol. 2025 Mar 27;13:1523593. doi: 10.3389/fcell.2025.1523593 (PMC11983436; doi:10.3389/fcell.2025.1523593)
Supplement: Supplementary file 1 [file DataSheet1.docx]

Supplementary Material

# Supplementary Figures


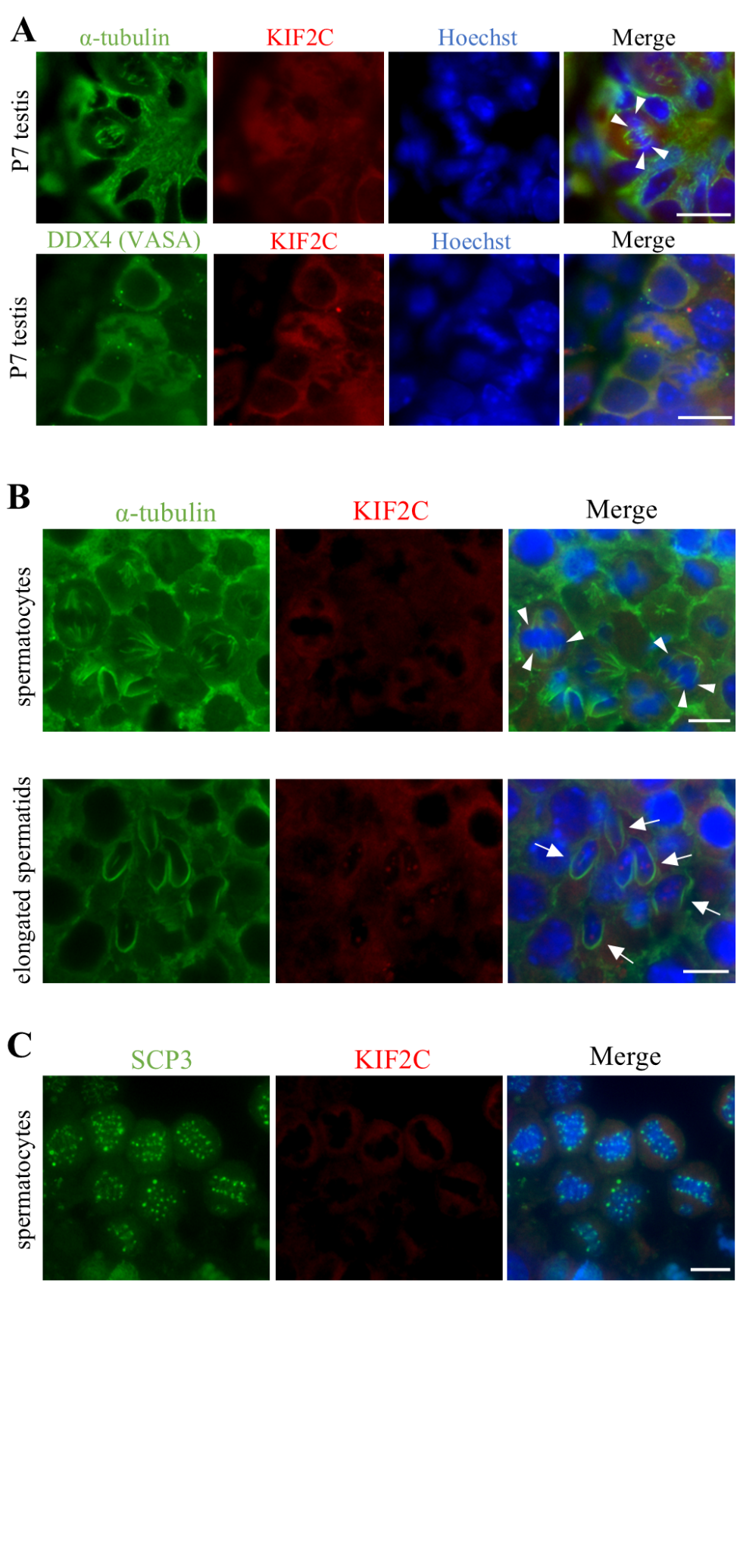


**Supplementary Figure 1.** KIF2C was not localized to centromeres in WT mice spermatogonia and *Kif2c* cKO mice spermatocytes. **(A)** The localization analysis of KIF2C in spermatogonia mitosis at PD7 WT mice testes using IHC (green: anti-α-tubulin, red: anti-KIF2C, blue: Hoechst 33342) (green: anti-VASA, red: anti-KIF2C, blue: Hoechst 33342). Arrowheads indicate spindle tip at metaphase spermatocytes. Scale bars = 10 µm. **(B)** Confirmation of KIF2C disappearance at centromere in spermatocyte and manchette in elongated spermatids in 8-week-old *Kif2c* cKO mice using IHC (green: anti-α-tubulin, red: anti-KIF2C, blue: Hoechst 33342). Arrowheads indicate spindle tip at metaphase spermatocytes. Arrows indicate the manchette in elongated spermatids. Scale bars = 50 µm (low magnification) and 10 µm (high magnification). (C) The localization analysis of KIF2C at meiotic metaphase in spermatocyte in 8-week-old *Kif2c* cKO mice using IHC (green: anti-SCP3, red: anti-KIF2C, blue: Hoechst 33342). Scale bars = 10 µm.


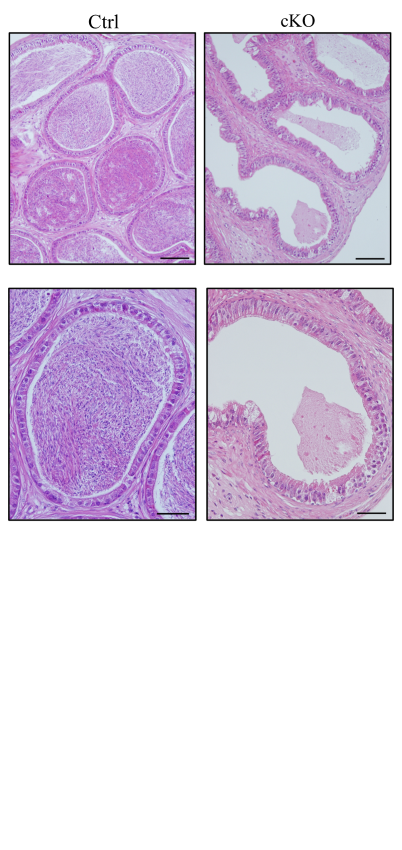


**Supplementary Figure 2.** No spermatozoa are present in cauda epididymis in *Kif2c* cKO mice. PAS-Hematoxylin staining of cauda epididymis. Scale bars = 100 µm (low magnification) and 50 µm (high magnification).
